# VPAC Receptor Subtypes Tune Purinergic Neuron-to-Glia Communication in the Murine Submucosal Plexus

**DOI:** 10.3389/fncel.2017.00118

**Published:** 2017-04-25

**Authors:** Candice Fung, Werend Boesmans, Carla Cirillo, Jaime P. P. Foong, Joel C. Bornstein, Pieter Vanden Berghe

**Affiliations:** ^1^Department of Physiology, The University of MelbourneParkville, VIC, Australia; ^2^Laboratory for Enteric Neuroscience (LENS), Translational Research Center for Gastrointestinal Disorders (TARGID), KU LeuvenLeuven, Belgium

**Keywords:** enteric nervous system, enteric glia, enteric circuitry, purinergic signaling, vasoactive intestinal peptide

## Abstract

The enteric nervous system (ENS) situated within the gastrointestinal tract comprises an intricate network of neurons and glia which together regulate intestinal function. The exact neuro-glial circuitry and the signaling molecules involved are yet to be fully elucidated. Vasoactive intestinal peptide (VIP) is one of the main neurotransmitters in the gut, and is important for regulating intestinal secretion and motility. However, the role of VIP and its VPAC receptors within the enteric circuitry is not well understood. We investigated this in the submucosal plexus of mouse jejunum using calcium (Ca^2+^)-imaging. Local VIP application induced Ca^2+^-transients primarily in neurons and these were inhibited by VPAC1- and VPAC2-antagonists (PG 99-269 and PG 99-465 respectively). These VIP-evoked neural Ca^2+^-transients were also inhibited by tetrodotoxin (TTX), indicating that they were secondary to action potential generation. Surprisingly, VIP induced Ca^2+^-transients in glia in the presence of the VPAC2 antagonist. Further, selective VPAC1 receptor activation with the agonist ([K15, R16, L27]VIP(1-7)/GRF(8-27)) predominantly evoked glial responses. However, VPAC1-immunoreactivity did not colocalize with the glial marker glial fibrillary acidic protein (GFAP). Rather, VPAC1 expression was found on cholinergic submucosal neurons and nerve fibers. This suggests that glial responses observed were secondary to neuronal activation. Trains of electrical stimuli were applied to fiber tracts to induce endogenous VIP release. Delayed glial responses were evoked when the VPAC2 antagonist was present. These findings support the presence of an intrinsic VIP/VPAC-initiated neuron-to-glia signaling pathway. VPAC1 agonist-evoked glial responses were inhibited by purinergic antagonists (PPADS and MRS2179), thus demonstrating the involvement of P2Y_1_ receptors. Collectively, we showed that neurally-released VIP can activate neurons expressing VPAC1 and/or VPAC2 receptors to modulate purine-release onto glia. Selective VPAC1 activation evokes a glial response, whereas VPAC2 receptors may act to inhibit this response. Thus, we identified a component of an enteric neuron-glia circuit that is fine-tuned by endogenous VIP acting through VPAC1- and VPAC2-mediated pathways.

## Introduction

The enteric nervous system (ENS) is located within the wall of the gastrointestinal tract and comprises a vast integrative network of neurons and glia organized in two concentric layers (Furness, 2012). The regulation of intestinal functions is finely driven by neurons and glia that interact via neurotransmitters. Though it is now well established that enteric glia are involved in the control of intestinal function, the nature of the signaling mechanisms operating within the neuro-glial circuitry remains largely unknown. Evidence for communication between enteric neurons and glia originates from ultrastructural studies demonstrating that nerve endings terminating on enteric glia have vesicle-containing presynaptic specializations (Gabella, 1981). In addition, active synaptic release sites surrounding enteric glia have been identified (Vanden Berghe and Klingauf, 2007). Enteric glia in turn express a variety of receptors for neurotransmitters (Rühl, 2005), including purinergic receptors that have been established to be important in neuron-glia communication (Gomes et al., 2009; Gulbransen and Sharkey, 2009; Boesmans et al., 2013a).

In addition to small signaling molecules utilized in neuro-neuronal communication, several peptides are involved in ENS signaling. Vasoactive intestinal peptide (VIP), one of the most abundant neuropeptides in the ENS, has long been considered a putative enteric neurotransmitter (Jessen et al., 1980). VIP-immunoreactive nerve terminals are present in both the submucosal (Foong et al., 2014) and myenteric plexuses (Sang and Young, 1996; Qu et al., 2008), and VIP depolarizes both submucosal (Mihara et al., 1985) and myenteric neurons (Palmer et al., 1987). VIP is a potent secretagogue and VIP neurons in the submucosal plexus regulate intestinal secretion (Banks et al., 2005; Burleigh and Banks, 2007; Fung et al., 2014). VIP is also found in interneurons of the myenteric plexus (Sang and Young, 1996; Sang et al., 1997), but its role in neurotransmission within the enteric circuitry is unclear. VIP acts via two main G-protein coupled receptors: VPAC1 and VPAC2 (Laburthe et al., 2002), but the roles of these VPAC receptors within the ENS are not well documented. This is largely due to a lack of available specific agonists and antagonists, and the literature detailing VIP antagonism in the gut is inconsistent. There are discrepancies between studies conducted in different species and intestinal regions, with the reported efficacy of VIP antagonists in inhibiting VIP-mediated or VIP-evoked secretion varying significantly (Cox and Cuthbert, 1989; Burleigh and Kirkham, 1993; Reddix et al., 1994; Banks et al., 2005). The reported effects of the same VIP antagonist have even differed between studies using similar protocols in the same animal (Mourad and Nassar, 2000; Banks et al., 2005; Kordasti et al., 2006). For our study, we have selected VPAC1- and VPAC2-agonists (Gourlet et al., 1997b; Tsutsumi et al., 2002) and antagonists (Gourlet et al., 1997a; Moreno et al., 2000; Banks et al., 2005), which have shown consistent effects, to examine their relative roles in the submucosal plexus.

Calcium imaging is widely used to study enteric network activity (Martens et al., 2014; Hennig et al., 2015). Indeed, it is the only viable method for assessing enteric glial cell activity (Boesmans et al., 2013b) given that glia communicate within their network via Ca^2+^ signals (Ochoa-Cortes et al., 2016; Grubisic and Gulbransen, 2017). Using Ca^2+^-imaging in *Wnt1-Cre;R26R-GCaMP3* mice, we serendipitously found that selective activation of neural VPAC1 receptors induced purinergic signaling from cholinergic enteric neurons to glia. Here we describe components of an enteric neuron-glia circuit which may be regulated by VIP via VPAC1 and VPAC2 receptors and purinergic signaling in the submucosal plexus of mouse small intestine.

## Materials and Methods

### Tissue Preparation and Ca^2+^-Imaging in Submucosal Plexus

*Wnt1-Cre;R26R-GCaMP3* mice (aged 6–13 weeks) of either sex were killed by cervical dislocation. All procedures are approved by the Animal Ethics Committees of the University of Leuven (Belgium) and the University of Melbourne (Australia). *Wnt1-Cre;R26R-GCaMP3* mice express the fluorescent Ca^2+^ indicator GCaMP3 in all enteric neurons and glia (Danielian et al., 1998; Zariwala et al., 2012). A segment of jejunum (approximately 5 cm long) was collected and immersed in 95% oxygen/5% carbon dioxide-bubbled Krebs solution (in mM: 120.9 NaCl, 5.9 KCl, 1.2 MgCl_2_, 1.2 NaH_2_PO_4_, 14.4 NaHCO_3_, 11.5 glucose and 2.5 CaCl_2_). The tissue was opened along the mesenteric border and pinned flat with mucosa up in a silicone elastomer-coated dish. Some tissues were immediately fixed in 4% formaldehyde/PBS for 45 min at room temperature for immunohistochemical staining. In others that were used for imaging, the submucosal plexus with the mucosa intact was separated from the underlying muscle layers by microdissection, and the tissue was stabilized by stretching the tissue with the plexus facing up over an inox ring, before clamping it with a rubber O-ring (Vanden Berghe et al., 2002). Up to five ring preparations were obtained from each jejunal segment.

Two microscopy setups were used to image the ring preparations. In Leuven (BE), a 20× (NA 1.0) water dipping lens was used on an upright Zeiss Examiner microscope (Carl Zeiss, Oberkochen, Germany) equipped with a monochromator (Poly V) and cooled CCD camera (Imago QE), both from TILL Photonics (Gräfelfing, Germany). Images (640 × 512) on this system were acquired at 2 Hz. In Melbourne (AU), the ring preparations were imaged via an Olympus 20× (NA 0.5) water dipping lens on an upright Zeiss Axioscope microscope with a Zeiss AxioCam MRm camera and images (278 × 278) were acquired at 1 Hz. Preparations were constantly superfused (1 ml/min) with 95% oxygen/5% carbon dioxide-gassed Krebs at room temperature.

Ganglia of interest were stimulated chemically or electrically. Agonists were applied to the preparations by pressure ejection (2 s duration; 9 psi) via a micropipette (tip diameter ~20 μm) positioned approximately 50 μm from the selected ganglion. For time control experiments, each agonist was applied three times separated by 5 min to ensure reproducible responses. For electrical stimulation experiments, trains of 300 μs pulses (2 s, 20 Hz) were applied by a focal stimulating electrode (tungsten; 50 μm diameter) on an internodal strand leading to the ganglion imaged. For time control experiments, ganglia were stimulated twice separated by 5 min.

Antagonist experiments consisted of two consecutive applications of agonist or two consecutive trains of electrical stimulation, with the antagonist superfused prior to the 2nd stimulation. Thus the 1st response is considered the control response. Antagonists were superfused via a local perfusion pipette. Each preparation was only exposed to a single antagonist once and to only one type of agonist. At least three animals were examined for each set of experiments.

After calcium imaging experiments, selected preparations were fixed in 4% formaldehyde/PBS for 45 min at room temperature for *post hoc* immunohistochemistry.

### Drugs

Agonists used included VIP (human, mouse, rat; Bachem, Bubendorf, Switzerland), VPAC1 agonist, [K15, R16, L27] VIP(1-2)/GRF(8-27) and VPAC2 agonist, BAY 55-9837 (both synthesized by Mimotopes, Clayton, VIC, Australia), and the P2Y1 agonist 2-methyl-thio-ADP (2MeSADP; Sigma Aldrich, Castle Hill, NSW, Australia). Superfusion of 1 μM VIP depolarized myenteric neurons (Palmer et al., 1987). Hence, 100 μM VIP was used to fill the micropipette for local application by pressure ejection, as the solution may be diluted up to approximately 100-fold in superfused preparations (Liu et al., 1997). The VPAC1 agonist [K15, R16, L27]VIP(1-2)/GRF(8-27) and the VPAC2 agonist BAY 55-9837 were also used at 100 μM, as VIP and [K15, R16, L27]VIP(1-2)/GRF(8-27) have similar potencies on the VPAC1 receptor (Gourlet et al., 1997b), while BAY 55-9837 has shown to be effective on hypothalamic neurons when superfused at 1–10 μM (Hermes et al., 2009; Pantazopoulos et al., 2010).

VIP antagonists used include the VPAC1 antagonist PG97-269, and the VPAC2 antagonist, PG 99-465 (both from Mimotopes). PG97-269 inhibits VIP-evoked intestinal secretion at 1 μM (Fung et al., 2014), while PG 99-465 inhibits VIP-evoked responses in hippocampal neurons at 100 nM–10 μM (Pakhotin et al., 2005; Cunha-Reis et al., 2014). Accordingly, we superfused each VPAC antagonist at a concentration of 1 μM. Other antagonists include tetrodotoxin (TTX; 1 μM; Acros, Geel, Belgium); hexamethonium bromide (200 μM; Sigma; Bornem, Belgium), pyridoxal-phosphate-6-azophenyl-2′,4′-disulfonic acid (PPADS; 30 μM; Sigma), and MRS2179 (10 μM; Tocris, Bristol, UK). The VPAC1- and VPAC2 antagonists were applied 5 min prior to agonist application as they have been shown to be most effective with short incubations (Pakhotin et al., 2005; Fang et al., 2006). TTX, hexamethonium, PPADS and MRS2179 were superfused for 10 min.

All drugs were prepared as stock solutions using water, except for TTX which was prepared in citrate buffer. TTX, 2MeSADP, and MRS2179 were stored at 4°C. Hexamethonium bromide, PPADS, VIP, [K15, R16, L27]VIP(1-2)/GRF(8-27), BAY 55-9837, PG97-269 and PG 99-465 were stored as aliquots at −20°C. Agonists and antagonists were diluted in Krebs to achieve the desired final concentration.

### Analysis

Only ganglia directly adjacent to the micropipette were considered for analysis. Neurons were identified by the size of their cell bodies (~20 μm diameter; Gabella and Trigg, 1984) and lack of fluorescence in their nuclei. Glia were identified by the size of their cell bodies (<5 μm diameter; Gabella and Trigg, 1984), and their morphology and location (either intraganglionic type I cells with irregular branched processes, or elongated type II cells within/at the edge of internodal strands; Boesmans et al., 2015). Ca^2+^-imaging analyses were performed with custom-written routines in Igor Pro (Wavemetrics, Lake Oswego, OR, USA). Regions of interest were drawn to calculate the average [Ca^2+^]_i_ signal intensity. Values were then normalized to the baseline fluorescence intensity (F_i_/F_o_). Responses were considered when the [Ca^2+^]_i_ signal increased above baseline by at least five times the intrinsic noise. [Ca^2+^]_i_ peaks were calculated for each response, with the peak amplitude taken as the maximum increase in [Ca^2+^]_i_ from baseline (ΔF_i_/F_o_). For time controls, the Δ_i_/F_o_ of the 2nd agonist exposure is presented as a percentage of the 1st (% Δ_i_/F_o_). Similarly, the Δ_i_/F_o_ evoked in the presence of antagonists is presented as a percentage of the 1st control agonist response. Responses that were observed with the 2nd agonist exposure, but not the 1st were not considered in this analysis. The total number of cells responding to each agonist exposure was also counted. The total number of cells responding to the 2nd agonist application was then similarly normalized to that of the 1st control response and presented as a percentage of the control.

### Immunohistochemistry

Immediately-fixed jejunal segments from mice of a C57Bl6 background including *Wnt1-Cre;R26R-GCaMP3* mice, and fixed submucosal ring preparations, were used for immunohistochemical staining. The mucosa and submucosa of immediately-fixed tissues were separated from the underlying muscle layers by microdissection. The mucosa of immediately-fixed preparations and fixed submucosal ring preparations was then removed and the preparations were incubated with a blocking buffer containing 4% donkey serum (Merck Millipore, Overijse, Belgium) and 0.5% triton X-100 (Sigma) in PBS overnight at 4°C. Preparations were subsequently incubated in primary antisera (Table 1) for 24–48 h at 4°C, washed in PBS (3 × 10 min) and then incubated in secondary antisera (Table 2) for 2 h at room temperature. Primary and secondary antisera were diluted in the blocking buffer. Preparations were washed in PBS (3 × 10 min) before mounting on slides with Citifluor (Citifluor Ltd., Leicester, UK) or Dakocytomation fluorescent mounting medium (Carpinteria, CA, USA).

**Table 1:** **Primary antibodies used for immunohistochemistry**.

Primary antibodies	Host	Dilution	Source	References
VPAC1R	Rabbit	1:1000	Pierce biotechnology	Barbarin et al. (2014)
ChAT	Goat	1:500	Chemicon	Foong et al. (2014)
Peripherin (C-19)	Goat	1:500	Santa cruz biotechnologies	Martens et al. (2014)
GFAP	Chicken	1:5000	Abcam	Desmet et al. (2014)
GFAP	Guinea pig	1:1000	Synaptic systems	Valtcheva et al. (2016)
Tyrosine hydroxylase	Sheep	1:1000	Millipore	Pelayo et al. (2014)
VAChT	Guinea pig	1:100	Millipore	Kaji et al. (2016)
CGRP	Goat	1:1000	AbD Serotec	Bergner et al. (2014)
Hu	Human	1:5000	Gift from Miles Epstein	Foong et al. (2014)

*Abbreviations: VPAC1R, vasoactive intestinal peptide receptor 1; ChAT, choline acetyltransferase; GFAP, glial fibrillary acidic protein; VAChT, vesicular acetylcholine transporter; CGRP, calcitonin gene-related peptide*.

**Table 2:** **Secondary antibodies used for immunohistochemistry**.

Secondary antibodies	Host	Dilution	Source	References
Anti-rabbit AMCA	Donkey	1:250	Jackson immuno labs	Boesmans et al. (2009)
Anti-rabbit AF594	Donkey	1:400	Molecular probes	Boesmans et al. (2009)
Anti-goat AF594	Donkey	1:1000	Molecular probes	Desmet et al. (2014)
Anti-sheep AF647	Donkey	1:500	Molecular probes	Foong et al. (2014)
Anti-sheep AF488	Donkey	1:400	Molecular probes	Foong et al. (2014)
Anti-chicken AF594	Donkey	1:1000	Molecular probes	Desmet et al. (2014)
Anti-guinea pig FITC	Donkey	1:100	Millipore	Wang et al. (2013)
Anti-human AF647	Donkey	1:500	Jackson immuno labs	Papini et al. (2015)
Anti-human AF488	Donkey	1:800	Jackson immuno labs	Gal-Or et al. (2016)

Fluorescently labeled preparations were viewed under an epifluorescence microscope (BX 41 Olympus, Olympus, Aartselaar, Belgium) equipped with UMNUAUV, U-MWIBA3 and U-MWIY2 filtercubes for visualizing blue, green and red probes, respectively. Images were acquired with an XM10 (Olympus) camera using Cell^F software. Pictures were adjusted for contrast and brightness before overlay and quantification. Confocal images were recorded using a Zeiss LSM780 confocal microscope (Cell Imaging Core, KU Leuven, Belgium) or Zeiss Pascal confocal microscope (Biological Optical Microscopy Platform, The University of Melbourne, Parkville, VIC, Australia).

### Data and Statistics

Data are presented as mean ± the standard error of the mean (SEM). One-way analysis of variance (ANOVA) followed by Dunnett’s *post hoc* test (where appropriate) were conducted to determine statistical significance, unless specified otherwise. *P* < 0.05 was considered significant. Analyses were performed using GraphPad Prism 5.0 (GraphPad softwares, San Diego, CA, USA).

## Results

### VIP Evokes Neuronal [Ca^2+^]_i_ Transients in Submucosal Plexus

**Figure 1:**
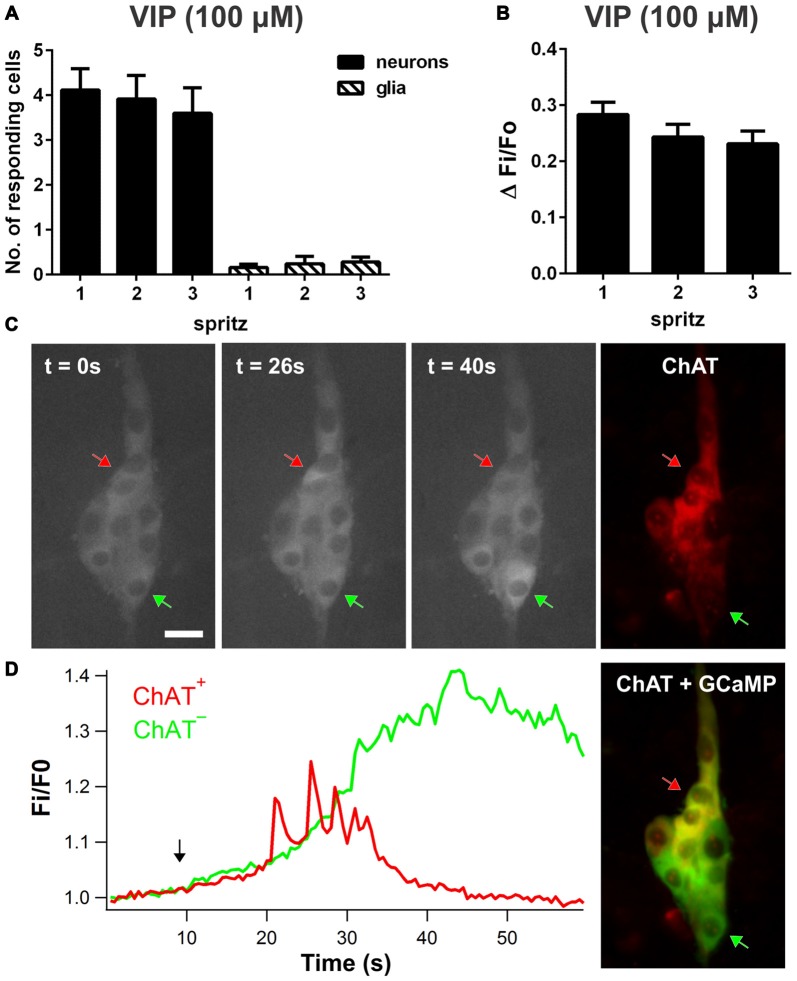
**Vasoactive intestinal peptide (VIP)-induced [Ca^2+^]_i_ transients in enteric neurons and glia. (A)** Number of submucosal neurons and glia responding per ganglion to three repeated local spritz-applications (2 s duration) of VIP (100 μM) separated by 5 min in time control experiments. **(B)** Maximum amplitudes of VIP-evoked neuronal [Ca^2+^]_i_ transients were reproducible in time control experiments. **(C)** Fluorescence images of ChAT^+^ (red arrow) and ChAT^−^ submucosal neurons (green arrow) displaying a [Ca^2+^]_i_ rise in response to VIP over time. Scale bar = 20 μm. Corresponding ChAT immunolabeling was used to identify ChAT^+^ and ChAT^−^ neurons. **(D)** Corresponding traces of VIP-induced [Ca^2+^]_i_ responses in selected ChAT^+^ and ChAT^−^ neurons. The black arrow in the graph indicates VIP application.

Local VIP (100 μM) application by pressure ejection evoked transient increases in intracellular Ca^2+^ concentration ([Ca^2+^]_i_) in neurons (Figure 1A); 44 ± 5% of neurons per ganglion responded with consistent [Ca^2+^]_i_ rises (ΔF_i_/F_o_: 0.28 ± 0.02, *n* = 103 neurons, 18 preparations; Figure 1B). Glial [Ca^2+^]_i_ transients were rarely observed (2/25 ganglia). Ganglia imaged in these experiments contained on average 9.3 ± 0.5 neurons. *Post hoc* immunohistochemistry was performed on some preparations to characterize the types of neurons which responded to VIP (100 μM; Figure 1C). Both ChAT^+^ neurons and ChAT^−^ neurons responded to VIP, but the characteristics of [Ca^2+^]_i_ transients observed in the two neuronal subtypes were distinctly different (Figure 1D). VIP-responsive ChAT^+^ neurons displayed [Ca^2+^]_i_ transients with a duration (at t50%; 50% of the peak amplitude) of 11.8 ± 0.8 s (*n* = 24 neurons), while ChAT^−^ neurons displayed slower responses with a significantly longer duration of 15.3 ± 1.0 s (*n* = 42 neurons; unpaired *t*-test, *P* = 0.016). The average latency of responses was 7.6 s ± 0.5 and ranged from 1.5 s to 18 s, but did not differ between ChAT^+^ and ChAT^−^ neurons.

**Figure 2:**
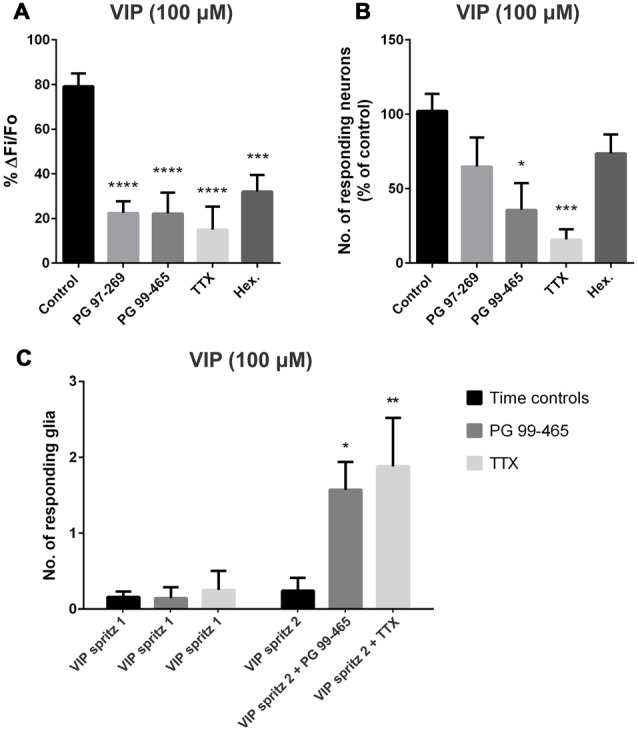
**VIP-evoked glial and neuronal responses in the presence of various antagonists. (A)** The selective VPAC1 antagonist (PG 97-269; 1 μM), the VPAC2 antagonist (PG 99-465; 1 μM), tetrodotoxin (TTX; 1 μM) and the nicotinic antagonist hexamethonium (Hex.; 200 μM) all significantly inhibited the peak amplitude of the VIP (100 μM)-evoked neuronal [Ca^2+^]_i_ transients (one-way analysis of variance (ANOVA) and Dunnett’s test, ***P < 0.001, ****P < 0.0001). **(B)** The VPAC2 antagonist also inhibited the number of neurons responding to VIP compared to control. TTX (1 μM) significantly inhibited the number of neurons responding to VIP and near abolished the neuronal response (one-way ANOVA and Dunnett’s test, *P < 0.05, ^***^P < 0.001). VIP-induced glial [Ca^2+^]_i_ transients were revealed by antagonists. The number of submucosal glia responding to a local spritz-application of VIP (100 μM) in the presence of **(C)** the VPAC2 antagonist PG 99-465 (1 μM; *n* = 7) or tetrodotoxin (TTX; Na^+^ channel blocker; 1 μM; *n* = 8) was significantly increased compared to control conditions (two-way ANOVA and Sidak’s test, *P < 0.05, **P < 0.01). Glial responses were not observed in time control experiments where VIP was applied twice under control conditions, separated by a 5 min washout (*n* = 25 preparations).

The VIP-evoked response was nearly abolished by the Na^+^-channel blocker TTX (1 μM), which significantly reduced the amplitude of the response (to 15 ± 10% of the control; one-way ANOVA and Dunnett’s test, *P* < 0.0001; *n* = 40 neurons, 8 preparations; Figure 2A) and the number of responding neurons per ganglion (to 16 ± 7% of the control; one-way ANOVA and Dunnett’s test, *P* < 0.001; Figure 2B). This suggests that the majority of [Ca^2+^]_i_ transients observed were secondary to action potential generation. Furthermore, reminiscent of the effect of the VPAC2 antagonist, TTX uncovered VIP-evoked glial responses, with 1.9 ± 0.6 glial cells responding per ganglion (*n* = 15 glia; Figure 2C).

As VPAC1 receptors have been previously shown to be expressed on many cholinergic submucosal neurons (Fung et al., 2014), we examined the involvement of cholinergic transmission in the response to VIP using the nicotinic receptor antagonist hexamethonium (200 μM). Blocking this major form of fast excitatory neurotransmission within the ENS reduced the amplitude of VIP-evoked responses (to 32 ± 7% of the control; one-way ANOVA and Dunnett’s test, *P* < 0.001; *n* = 24 neurons, 6 preparations; Figure 2A). In contrast to TTX and the VPAC2 antagonist (see below), hexamethonium did not reveal VIP-evoked glial responses.

### Inhibiting VPAC2 Receptors Reveals Responses to VIP in Glia

We next examined the relative contributions of VPAC1 and VPAC2 receptors to the VIP-induced [Ca^2+^]_i_ transients using the VPAC1 antagonist PG 97-269 (Banks et al., 2005) and the VPAC2 antagonist PG 99-465 (Moreno et al., 2000; Dickson et al., 2006). The VIP (100 μM)-evoked neuronal response amplitudes were significantly reduced in the presence of the VPAC1 antagonist PG 97-269 (1 μM; to 22 ± 5% of the control; one-way ANOVA and Dunnett’s test, *P* < 0.0001; *n* = 47 neurons, 9 preparations; Figure 2A), but the number of neurons responding did not change (Figure 2B). The VPAC2 antagonist PG 99-465 (1 μM) effectively reduced both the amplitude of the VIP-evoked response (to 22 ± 9% of the control; one-way ANOVA and Dunnett’s test, *P* < 0.0001; *n* = 30 neurons, 7 preparations; Figure 2A) and the number of responding neurons per ganglion (to 36 ± 18% of the control; one-way ANOVA and Dunnett’s test, *P* < 0.05; Figure 2B). These data indicate that the VIP-evoked [Ca^2+^]_i_ transients in neurons involve both VPAC1 and VPAC2 receptor activation. Interestingly, in 6/7 preparations, glial responses to VIP were uncovered in the presence of the VPAC2 antagonist, where a [Ca^2+^]_i_ rise was observed in 1.6 ± 0.4 glial cells per ganglion (*n* = 11 glial cells; Figure 2C).

### Selective VPAC1 Activation Predominantly Evokes [Ca^2+^]_i_ Transients in Enteric Glia

**Figure 3:**
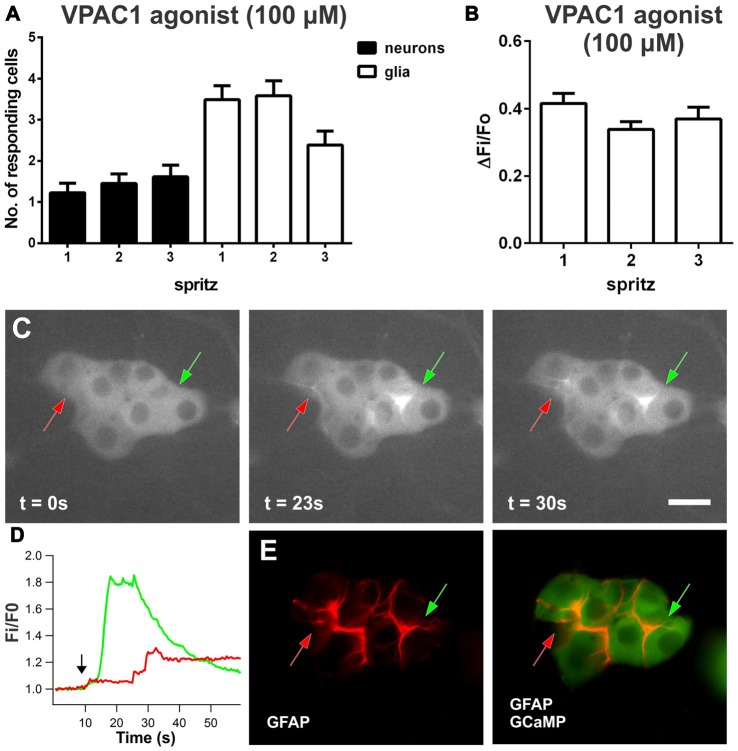
**VPAC1-induced [Ca^2+^]_i_ transients in enteric neurons and glia. (A)** Number of submucosal neurons and glia responding to three repeated local spritz-applications of VPAC1 agonist ([K15, R16, L27]VIP(1-7)/GRF(8-27); 100 μM) separated by 5 min in time control experiments. **(B)** Maximum amplitudes of VPAC1 agonist-evoked glial [Ca^2+^]_i_ transients were reproducible over time control experiments. **(C)** Glial fibers in a submucosal ganglion responding to VPAC1 agonist over time, as indicated by arrows. Scale bar = 20 μm.** (D)** VPAC1 agonist-induced [Ca^2+^]_i_ responses of selected glial fibers as indicated by color-coded arrows in **(C)** The black arrow in the graph marks the application of VPAC1-agonist. **(E)** Responding glial fibers identified by glial fibrillary acidic protein (GFAP) immunofluorescent labeling.

As inhibiting VPAC2 receptors unveiled glial [Ca^2+^]_i_ transients in response to VIP, we investigated whether the VIP-induced glial response is mediated via VPAC1 receptors. Local spritz application of [K15, R16, L27]VIP(1-2)/GRF(8-27; VPAC1 agonist; 100 μM; Gourlet et al., 1997b) to submucosal ganglia predominantly evoked [Ca^2+^]_i_ responses in glial cells (Figure 3A). By contrast, BAY 55-9837 (VPAC2 agonist; 100 μM) did not evoke reproducible [Ca^2+^]_i_ responses in neurons or glia. The VPAC1 agonist evoked [Ca^2+^]_i_ transients in 3.5 ± 0.4 glia per ganglion (Figure 3A) with a peak amplitude (ΔF_i_/F_o_) of 0.42 ± 0.03 (*n* = 108 glial cells, 22 preparations; Figure 3B). With *post hoc* immunohistochemistry for glial fibrillary acidic protein (GFAP), we confirmed that the fibers activated in response to the VPAC1 agonist were glial (Figures 3C–E). The types of glial cells responding were classified based on their location and morphology (Boesmans et al., 2015). We found that predominantly type I enteric glial cells responded to VPAC1 agonist application: 2.1 ± 0.3 type I vs. 1.4 ± 0.2 type II cells displayed [Ca^2+^]_i_ transients per ganglion (paired *t*-test, *P* = 0.03; *n* = 31 ganglia).

The VPAC1 agonist also evoked some neuronal [Ca^2+^]_i_ responses (ΔF_i_/F_o_: 0.65 ± 0.1; *n* = 38 neurons, 22 preparations), where 13 ± 3% of neurons responded per ganglion. In this set of experiments, the mean number of neurons contained per ganglion imaged was 10.6 ± 0.6. Examining the time course of the VPAC1 agonist-evoked responses, some neuronal responses preceded glial activation and, interestingly, some neuronal responses were observed following the onset of a [Ca^2+^]_i_ rise in glia. Neurons that responded before glia displayed [Ca^2+^]_i_ transients with a latency of 4.3 ± 1.2 s (*n* = 10 neurons). Glial responses had a latency of 14 ± 1.0 s (*n* = 108 glia) and neurons that responded following glial activation had a latency of 19 ± 2 s (*n* = 28 neurons).

### VPAC1 Agonist-Evoked Glial Activation Involves a TTX-Insensitive Mechanism

**Figure 4:**
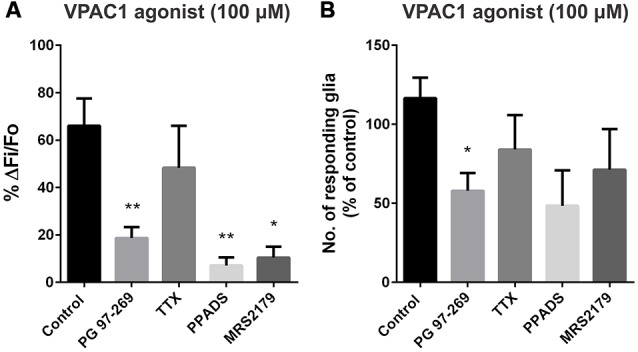
**VPAC1 agonist-evoked glial and neuronal [Ca^2+^]_i_ responses in the presence of antagonists. (A)** The peak amplitude of the VPAC1 agonist ([K15, R16, L27]VIP(1-7)/GRF(8-27); 100 μM)-evoked response was significantly inhibited by the selective VPAC1 antagonist (PG 97-269; 1 μM; one-way ANOVA and Dunnett’s test, **P < 0.01), but was not affected by TTX (1 μM). The P2 antagonist pyridoxal-phosphate-6-azophenyl-2’,4’-disulfonic acid (PPADS; 30 μM) and the selective P2Y1 antagonist MRS2179 (10 μM) were also effective in inhibiting the VPAC1 agonist response amplitude (one-way ANOVA and Dunnett’s test, *P < 0.05, **P < 0.01). **(B)** Only the VPAC1 antagonist significantly reduced the number of glia responding to the VPAC1 agonist (one-way ANOVA and Dunnett’s test, *P < 0.05).

We used PG-97-269 (VPAC1 antagonist; 1 μM) to test the specificity of the VPAC1 agonist (100 μM), and it significantly reduced the number of responding glia per ganglion (to 58 ± 11% of the control; one-way ANOVA and Dunnett’s test, *P* < 0.05; Figure 4B), as well as the peak amplitude of the glial [Ca^2+^]_i_ transients (to 19 ± 5% of the control; one-way ANOVA and Dunnett’s test, *P* < 0.01; *n* = 50 glial cells, 13 preparations; Figure 4A). However, the VPAC1 agonist-induced glial response was unaffected by TTX (1 μM; *n* = 24 glial cells, 5 preparations; Figure 4).

### The VPAC1 Receptor Is Expressed on Cholinergic Submucosal Neurons and Nerve Fibers

**Figure 5:**
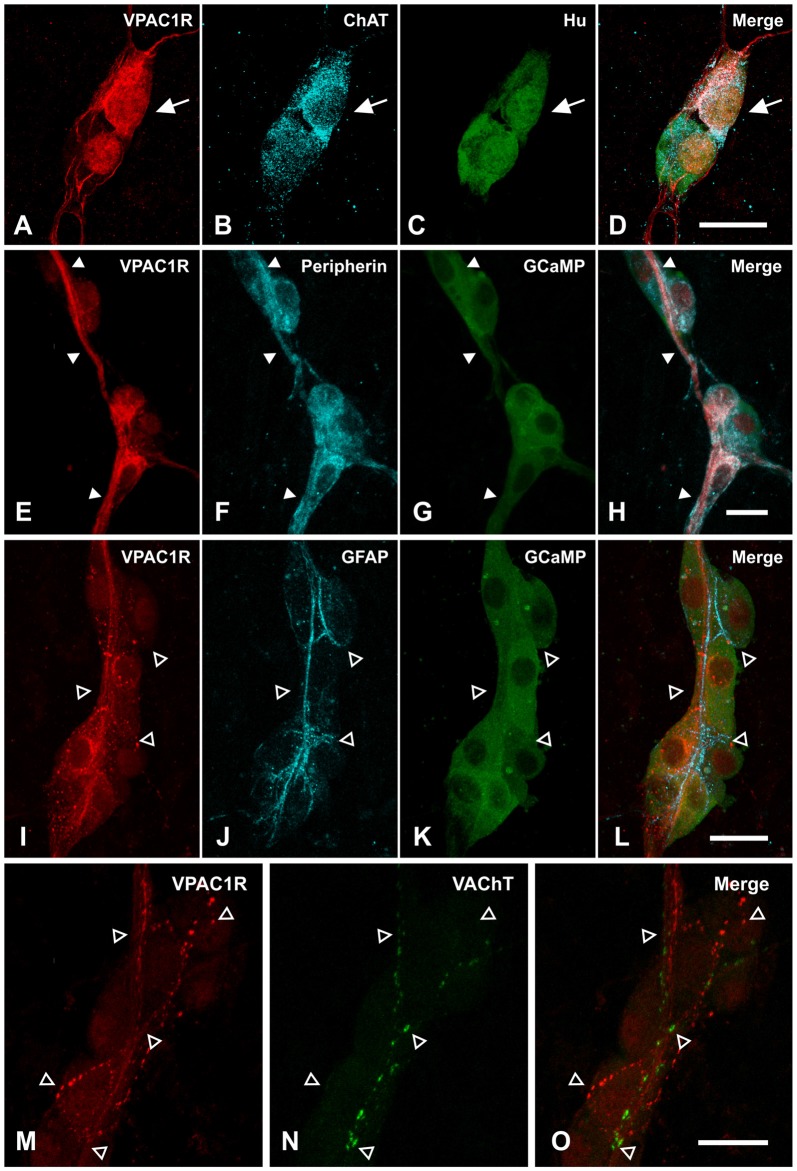
**Localizing VPAC1 receptor (VPAC1R)-expression in the submucosal plexus of mouse jejunum. (A–D)** Confocal micrographs demonstrating that VPAC1R-immunoreactive cell bodies were cholinergic, as marked by **(B)** ChAT and **(C)** Hu staining and indicated by the arrow. **(E–H)** VPAC1R expressing fibers also overlapped with peripherin^+^ nerve fibers (indicated by filled arrowheads). **(I–L)** However, VPAC1R did not colocalize with the GFAP^+^ glial fibers (open arrowheads). **(A–L)** are maximum projections of stacks of confocal images. **(M–O)** Confocal images taken at a single optical plane showing that VPAC1R- and Vesicular acetyltransferase (VAChT)-immunoreactivity also did not overlap (open arrowheads). Scale bars = 20 μm.

Given that the VPAC1 agonist-evoked glial response was TTX-insensitive, we next assessed whether enteric glia express VPAC1 receptors. Localization of the VPAC1 receptor was examined using immunohistochemistry (Figure 5). VPAC1 receptor expression was specifically neural, with the antisera labeling a subset of submucosal neurons and neuronal, but not glial, processes. VPAC1 receptor-immunoreactivity was observed in 12 ± 2% Hu^+^ cells and these were predominantly ChAT^+^ (93 ± 4%; *n* = 284 neurons; 3 animals; Figures 5A–D). Some background nuclear labeling was observed in neurons, but this staining was not considered in the analysis. VPAC1 receptor staining also colocalized with peripherin, a label for neuronal fibers (Figures 5E–H; *n* = 3 animals). However, VPAC1 receptors did not colocalize with GFAP^+^ glial processes (*n* = 3 animals; Figures 5I–L). Since we found that VPAC1 receptors are expressed on some ChAT^+^ neurons, we also examined whether VPAC1 receptors may also be expressed on cholinergic varicosities by co-labeling for vesicular acetyltransferase (VAChT). However, there was no apparent overlap between VPAC1 and VAChT (*n* = 2 animals; Figures 5M–O).

**Figure 6:**
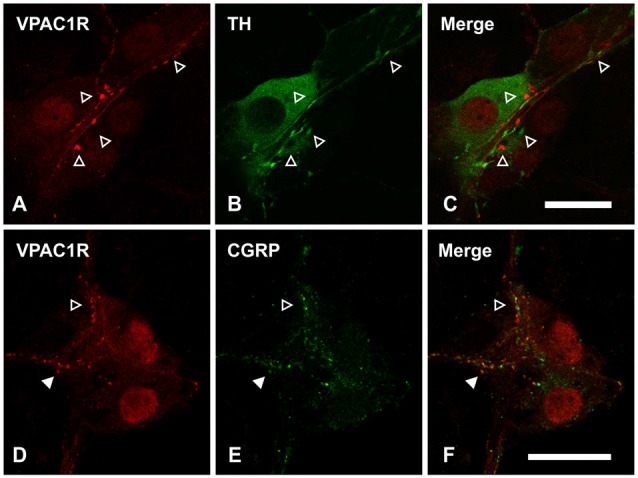
**Confocal images of VPAC1 receptor (VPAC1R)-immunoreactive nerve fibers and varicosities in the submucosal plexus of mouse jejunum taken at a single optical plane. (A–C)** VPAC1R did not colocalize with tyrosine hydroxylase (TH), as indicated by open arrowheads. **(D–F)** Confocal images of VPAC1 receptor (VPAC1R)- and calcitonin related-gene peptide (CGRP)-immunoreactive varicosities in a submucosal ganglion. Some overlap between VPAC1R- and CGRP-labeling was observed (filled arrowhead). However, not all VPAC1R^+^ varicosities were CGRP^+^ and vice versa (open arrowhead). Scale bars = 20 μm.

As it has been reported that enteric glia receive innervation from sympathetic fibers in guinea pig myenteric plexus (Gulbransen et al., 2010), we addressed whether VPAC1 receptors may also be expressed on sympathetic adrenergic nerve fibers. However, we did not observe any colocalization between tyrosine hydroxylase (TH; labels adrenergic nerves) and VPAC1 receptor expression (*n* = 2 animals; Figures 6A–C). In addition to TH nerve fibers, many submucosal neurons of mouse ileum are surrounded by calcitonin gene-related peptide (CGRP)-immunoreactive varicosities (Mongardi Fantaguzzi et al., 2009). Some colocalization of VPAC1 receptors with CGRP-immunoreactive varicosities was observed (*n* = 2 animals; Figures 6D–F).

### Inhibiting VPAC2 Receptors Reveals Delayed Glial Responses to Electrical Stimulation

To confirm the presence of a VIP/VPAC neuron-to-glia signaling pathway, we stimulated fiber tracts electrically to release endogenously-produced VIP. In these experiments, trains of stimuli evoked neuronal [Ca^2+^]_i_ transients (ΔF_i_/F_o_: 0.80 ± 0.08; *n* = 86 neurons) that were unaffected by the VPAC2 antagonist PG 99-465 (1 μM; *n* = 115). However, this antagonist revealed delayed glial responses to electrical stimulation in 7/13 preparations, where 1.3 ± 0.2 glia responded per ganglion. These delayed glial responses had a latency of 20.5 ± 2.9 s (*n* = 10 glial cells) and were not observed in time controls.

### VPAC1R Activation Stimulates Glial [Ca^2+^]_i_ Responses Via Purinergic Signaling

Purinergic signaling is important for enteric neuron-glia communication (Gomes et al., 2009; Gulbransen and Sharkey, 2009), so we examined the effect of purinergic antagonists on the VPAC1 agonist-stimulated glial responses. The P2 receptor antagonist PPADS (30 μM), significantly reduced the peak amplitude of the VPAC1 agonist-induced glial [Ca^2+^]_i_ response to 7 ± 3% of the control (one-way ANOVA and Dunnett’s test, *P* < 0.01; *n* = 31 glial cells, 6 preparations; Figure 4A). We then tested a specific P2Y1 antagonist, as P2Y1 receptors are associated with enteric glial activation (Gomes et al., 2009; Gulbransen et al., 2012). The P2Y1-selective antagonist MRS2179 (10 μM) significantly inhibited the amplitude of the VPAC1 agonist-induced glial response (to 10 ± 5% of the control; one-way ANOVA and Dunnett’s test, *P* < 0.05; *n* = 20 glial cells, 5 preparations; Figure 4A).

**Figure 7:**
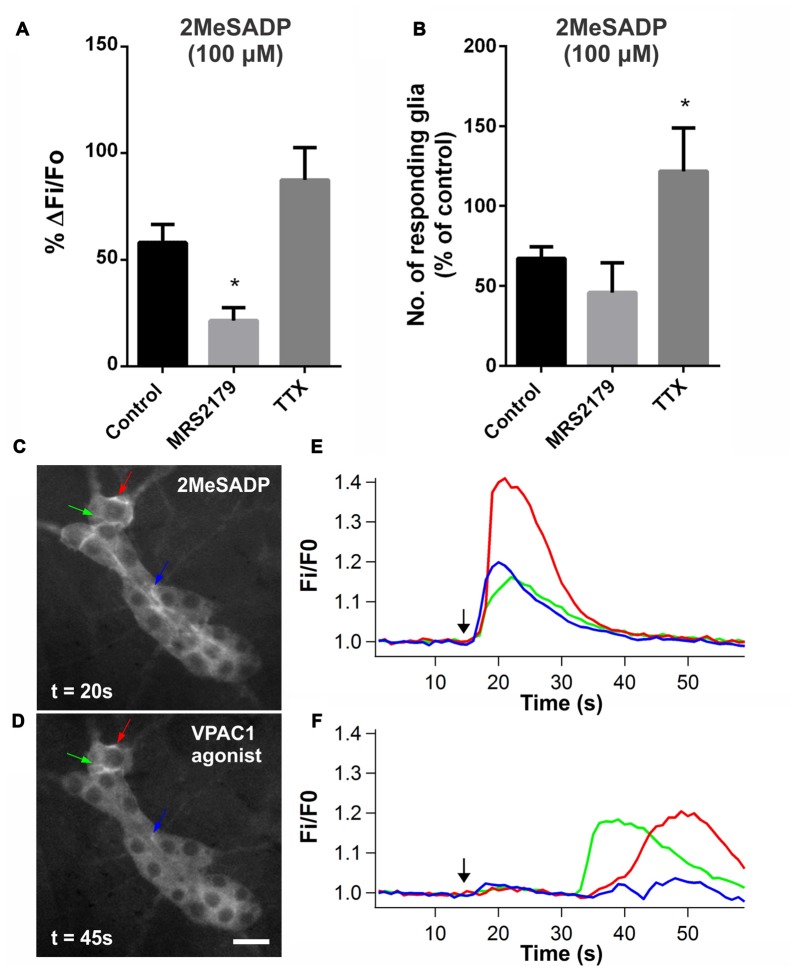
**P2Y1 agonist 2-methyl-thio-ADP (2MeSADP)-evoked glial [Ca^2+^]_i_ responses. (A)** The P2Y1 antagonist MRS2179 (10 μM) significantly inhibited the amplitude of glial cells responding to 2MeSADP (100 μM; one-way ANOVA and Dunnett’s test, *P < 0.05). **(B)** The number of glia responding to 2MeSADP was increased in the presence of TTX (1 μM) compared to control (one-way ANOVA and Dunnett’s test, *P < 0.05). **(C–F)** Some glial cells displayed [Ca^2+^]_i_ transients in response to both 2MeSADP (100 μM) and VPAC1 agonist ([K15, R16, L27]VIP(1-7)/GRF(8-27); 100 μM), as indicated by arrows. However, the latency of the 2MeSADP-evoked glial [Ca^2+^]_i_ responses was significantly shorter than that of the VPAC1 agonist. The black arrows in the graphs mark the application of agonist.

To further investigate the role of P2Y1 receptors, we examined the response to spritz-applied 2MeSADP (100 μM; P2Y1-specific agonist). Consistent with observations in mouse myenteric plexus (Brown et al., 2016), stimulation with 2MeSADP primarily evoked [Ca^2+^]_i_ transients in submucosal glial cells. 2MeSADP evoked responses in 4.8 ± 0.4 glial cells per ganglion (ΔF_i_/F_o_: 0.16 ± 0.02; *n* = 43 glial cells, 6 preparations). We tested the specificity of 2MeSADP and found that the peak amplitude of the response was significantly reduced by MRS2179 (10 μM; to 21 ± 6% of the control; one-way ANOVA and Dunnett’s test, *P* < 0.05; *n* = 29 glial cells, 5 preparations; Figure 7A). TTX (1 μM) did not inhibit 2MeSADP-evoked glial responses, rather the number of glia responding was increased (to 122 ± 27% of control; one-way ANOVA and Dunnett’s test, *P* < 0.05; *n* = 6 preparations; Figure 7B).

We tested if agonists for the two receptors evoke [Ca^2+^]_i_ signals in the same glial cells to examine whether P2Y1 receptors act within the same pathway as that activated by VPAC1 receptors. We compared the [Ca^2+^]_i_ responses to 2MeSADP (100 μM) and VPAC1 agonist (100 μM) by sequentially applying these agonists to same ganglion, with each application separated by a 5 min washout. 2MeSADP evoked [Ca^2+^]_i_ responses in significantly more glia per ganglion than the VPAC1 agonist (4.6 ± 0.4 vs. 2.8 ± 0.5 respectively; paired *t*-test, *P* = 0.027; *n* = 10 ganglia, 7 preparations), and responses were unaffected by the order of agonist application. Notably, some glial cells responded to both agonists (Figures 7C,D), but 2MeSADP induced higher [Ca^2+^]_i_ peak amplitudes (ΔF_i_/F_o_: 0.23 ± 0.02 vs. 0.18 ± 0.02 respectively; paired *t*-test, *P* = 0.026; *n* = 24 glial cells). Moreover, the time course of the responses to the two agonists differed drastically (Figures 7E,F). The 2MeSADP-evoked glial response was rapid and short-lived with a latency of 2.8 ± 0.2 s, while the VPAC1 agonist glial response exhibited a significant delay with a latency of 16.3 ± 2.1 s (paired *t*-test, *P* < 0.0001; *n* = 24 glial cells).

## Discussion

In this study, we identified a hitherto unknown purinergic neuro-glia interaction initiated by selective VPAC1-activation in the mouse submucosal plexus. VIP and the VPAC1 agonist evoked distinctly different [Ca^2+^]_i_ responses, where VIP evoked neuronal responses, and the VPAC1 agonist primarily evoked glial responses. Our data indicates that both VIP and the VPAC1 agonist initiate responses through neuronal activation. This is in agreement with previous reports showing that VIP depolarizes enteric neurons (Mihara et al., 1985; Palmer et al., 1987). Our study is the first to demonstrate that VIP, acting through neuronal VPAC1 receptors, initiates a sequential activation of neurons and glia. This pathway is in part inhibited by VPAC2 receptor activation.

While the direct cellular activity evoked by VIP (i.e., elevated cAMP) may not be visible, its effects on the neuron-glial circuitry can be examined using Ca^2+^ imaging. This definitely holds true in the cells further down the circuitry that may be excited by synaptic mechanisms. Even neurons expressing VPAC receptors in which cAMP was first increased may display—albeit delayed—rises in [Ca^2+^]_i_ (Shah et al., 2008; Langer, 2012; Li et al., 2013). In the case of classic electrical excitation, it has been shown that action potential firing correlates well with increases in [Ca^2+^]_i_ (Vanden Berghe et al., 2001; Michel et al., 2011; Martens et al., 2014). [Ca^2+^]_i_ transients in enteric neurons are mediated by various Ca^2+^ channels including N-, P/Q-, L- and R-type Ca^2+^ channels (Garaulet et al., 1996; Starodub and Wood, 1999; Reis et al., 2000; Smith et al., 2003; Bian et al., 2004; Bian and Galligan, 2007; Hao et al., 2011). [Ca^2+^]_i_ signals that we observe with GCaMP3 are unlikely to directly correspond with slow EPSPs. Rather, it is when the slow excitatory synaptic input initiates action potential firing that accompanying [Ca^2+^]_i_ signals can be resolved (Shuttleworth and Smith, 1999; Tack and Smith, 2004). Indeed, the neuronal [Ca^2+^]_i_ response to VIP was largely inhibited by TTX and hexamethonium, indicating that much of response was secondary to action potential generation and synaptic communication. This may also contribute to the explanation for the slow onset of neuronal [Ca^2+^]_i_ responses observed following VIP application, where the mean latency was 7 s and ranged from 1.5 s to 18 s. Furthermore, the response kinetics of GCaMP3 are slow relative to electrical activity (Rakhilin et al., 2016). Additionally, electrophysiological studies have reported electrically-evoked slow EPSPs with latencies ranging from 0.3 s to 12 s in AH neurons (Johnson and Bornstein, 2004). Dual impalement studies of pairs of S-type submucosal neurons have also shown that evoking action potentials in a presynaptic neuron can induce a slow postsynaptic depolarization in a postsynaptic neuron with a latency ranging from 14 s to 35 s (Reed and Vanner, 2001).

In neurons, both VPAC antagonists inhibited the VIP-evoked increase in [Ca^2+^]_i_, which indicates that both VPAC receptor subtypes are involved in mediating this response. The VPAC1- and VPAC2 antagonists each reduced the VIP-evoked [Ca^2+^]_i_ response amplitudes by 78%, which suggests that there is a degree of overlap between the expression of these two receptor subtypes. Alternatively, activation of these receptors excites different elements within the circuitry that converge on to the same pathway. Despite the ability of the VPAC1 antagonist to reduce VIP-evoked neuronal response amplitudes by 78%, the VPAC1 agonist only evoked a minor neuronal response with only 1 neuron responding per ganglion (roughly 10% of neurons) on average. This is unsurprising considering that the VPAC1 antagonist did not significantly inhibit the number of neurons responding to VIP, whereas the VPAC2 antagonist did. This suggests that neurons receiving cholinergic input from VPAC1-expressing neurons also require VPAC2 receptor activation to produce significant [Ca^2+^]_i_ transients. Furthermore, the minor neuronal response is also consistent with the finding that VPAC1-expressing neurons comprise a small proportion of the total number of submucosal neurons (~12%).

While VIP alone did not evoke [Ca^2+^]_i_ transients in glia, glial responses were revealed with VPAC2 receptor antagonism and by blocking TTX-sensitive Na^+^ channels. The discrepancy in [Ca^2+^]_i_ responses to VIP and the VPAC1 agonist may be accounted for by a VPAC2 receptor-mediated and TTX-sensitive inhibitory pathway which suppresses VPAC1-mediated glial responses. Interestingly, delayed glial responses were also observed following trains of electrical stimuli when VPAC2 receptors were inhibited. This suggests that VPAC2 receptors are involved in inhibiting glial activation by endogenously released mediators, presumably VIP.

Blocking nicotinic transmission, a major form of excitatory neurotransmission in the ENS (Galligan and North, 2004; Gwynne and Bornstein, 2007; Foong et al., 2015), with hexamethonium reduced the number of neurons responding to VIP. This suggests that many neurons respond secondarily to activation of cholinergic neurons and is consistent with the finding that cholinergic submucosal neurons express VPAC1 receptors. Indeed, both the VPAC1 antagonist and hexamethonium appear to inhibit the VIP-evoked response to a similar extent. Taken together, this data suggests that cholinergic transmission mediates the VPAC1 response in many neurons. However, unlike TTX and the VPAC2 antagonist, hexamethonium did not reveal glial responses. Thus, nicotinic neurotransmission and the neurons responding secondarily to VIP, do not appear to be involved in this neuron-glia interaction. This may suggest that the neural VPAC2-mediated inhibitory pathway is monosynaptic. Another possibility is that VPAC1 and VPAC2 may be co-expressed on the same neuron, and VPAC2-activation antagonizes the VPAC1-initiated signal.

There are some inconsistencies regarding the role of the VPAC2 receptor, as the VPAC2 agonist alone did not evoke [Ca^2+^]_i_ signals in neurons or glia. Perhaps this VPAC2 agonist BAY 55-9837 is ineffective in this system, as data obtained using the VPAC1 agonist and antagonist, and the VPAC2 antagonist, all suggest involvement of VPAC2 receptors in the VIP-evoked responses. Alternatively, the VPAC2 agonist may not induce a robust [Ca^2+^]_i_ rise, but rather preferentially activate the cAMP secondary messenger system (Dickson et al., 2006; Langer, 2012). Further, the lack of neuronal [Ca^2+^]_i_ responses to the VPAC2 agonist, despite the inhibitory effect of the VPAC2 antagonist on VIP-evoked neuronal [Ca^2+^]_i_ responses, may be explained if these [Ca^2+^]_i_ responses require activation of both VPAC1 and VPAC2 receptors. While the VPAC1 receptor agonist and antagonist used in this study have been assessed by others and are deemed specific (Dickson et al., 2006; Dickson and Finlayson, 2009), there is yet to be a highly specific VPAC2 receptor antagonist available (Dickson et al., 2006). Indeed, the VPAC2 antagonist PG 99-465 may also act at VPAC1 and pituitary adenylate cyclase activating peptide (PACAP)-specific PAC1 receptors (Dickson et al., 2006). Interestingly, these non-specific effects were only observed in a [cAMP]_i_ assay, but not a [Ca^2+^]_i_ assay (Dickson et al., 2006). However, the effects that we observed with each agonist and antagonist in this study were clearly distinct. The VPAC1 agonist mainly evoked glial responses. When VPAC2 receptors were blocked, VIP-evoked glial [Ca^2+^]_i_ responses were revealed, presumably through activating VPAC1 receptors. It is also possible that other receptors are activated by VIP to suppress the glial component of the VPAC1 receptor-mediated response, such as VPAC1 and VPAC2 receptor splice variants (Dickson and Finlayson, 2009). VPAC receptor oligomerization and interaction of VPAC receptors with accessory proteins (Couvineau and Laburthe, 2012) have also been reported, although the functional roles of these are unclear.

VPAC1 receptor expression on astrocytes has been shown in the central nervous system (CNS; Joo et al., 2004), but we found no evidence for VPAC1 expression on enteric glia as it did not colocalize with the glial marker GFAP. Since GFAP is part of the cytoskeleton, antibody labeling does not necessarily reveal the entire cytosol. Thus, we cannot fully exclude VPAC1 expression on the peripheral processes of glia that are not labeled by GFAP based on immunohistochemical approaches (Haseleu et al., 2013). This raises the possibility of VPAC1 receptors activating purinergic paracrine and/or autocrine signaling in glia. However, VPAC1 expression clearly colocalizes with the neuronal markers Hu and peripherin, which strongly suggests that its expression in the ENS is exclusively neural. Further, despite the finding that VPAC1 agonist-induced glial responses were TTX-insensitive, the long latency of these responses also suggests that glial responses are secondary to neuronal activation. These data perhaps suggest the involvement of TTX-resistant Na^+^ channels (Mao et al., 2006; Smith et al., 2012), and/or an action potential-independent mechanism. It is possible that the glial response is evoked by activating VPAC1 receptors on nerve fibers in contact with glia. As in the guinea pig, VPAC1 receptor expression in mouse jejunum was predominantly localized to cholinergic submucosal neurons (Fung et al., 2014), but VPAC1-immunoreactive varicosities did not overlap with VAChT in this mouse study. Perhaps this is not surprising, as it has been previously shown that some nerve terminals of ChAT-containing cell bodies may not necessarily be “cholinergic” in that they do not all contain VAChT (Sharrad et al., 2013). We also considered whether VPAC1 receptors may be expressed on sympathetic nerve fibers, as these innervate glia in the guinea pig myenteric plexus (Gulbransen et al., 2010). However, we found that VPAC1 receptors were not expressed on TH-containing fibers. Instead, we observed partial colocalization between CGRP- and VPAC1 receptors in varicosities. These CGRP^+^ nerve terminals may originate from extrinsic primary afferents (De Jonge et al., 2003), myenteric intrinsic sensory neurons (Qu et al., 2008), or submucosal CGRP neurons (Mongardi Fantaguzzi et al., 2009). However, the specific subtype/s expressing VPAC1 receptors, and whether extrinsic inputs are involved in this neuron to glia signaling pathway, require further investigation.

The mechanism involved in the TTX-insensitive VPAC1-mediated glial response remains unclear. However, TTX-insensitive P2X7-mediated enteric neuron-glia signaling has been previously reported in the mouse colon myenteric plexus, and was shown to involve neuronal ATP-release through pannexin-1 channels (Gulbransen et al., 2012). It is possible that pannexin-1 channels are similarly involved in the VPAC1 signaling pathway. Purines acting on P2 receptors is one of the major signaling pathways involved in neuron-glia transmission (Gulbransen and Sharkey, 2009; Boesmans et al., 2013b) and the most extensively studied. In accordance with these findings, we observed that the VPAC1 agonist-evoked glial response was largely inhibited by the P2 antagonist PPADS and also partially inhibited by the P2Y1-selective antagonist MRS2179. In further examining the P2Y1 component of this response, we demonstrated that glial cells which responded to the VPAC1 agonist could also be activated by the P2Y1 agonist 2MeSADP. Notably, the glial responses to the VPAC1 agonist occurred with a significant delay compared to those evoked by 2MeSADP. This is consistent with the notion that VPAC1 receptor activation first stimulates a neuron, or at least a neuronal release site, which then activates glia. Furthermore, the timing of responses indicates that P2Y1 receptors are most likely expressed on glial cells and that the 2MeSADP response results from a direct stimulation of these receptors. This is also compatible with our data showing that the 2MeSADP response was not inhibited by TTX. In fact, the number of glia responding to 2MeSADP was increased by TTX, suggesting either a 2MeSADP-mediated or an ongoing neural suppression of [Ca^2+^]_i_ signaling activity in glia. While studies in guinea pig have shown that functional P2Y1 receptors are expressed in submucosal and myenteric neurons (Hu et al., 2003; Gao et al., 2006), and 2MeSADP (10 μM) has been reported to evoke [Ca^2+^]_i_ responses in rat submucosal neurons (Christofi et al., 2004), in our hands 2MeSADP predominantly evoked [Ca^2+^]_i_ responses in glia. This may be due to species differences as our findings are in agreement with other studies in mice demonstrating that the activation of P2Y1 receptors primarily stimulates enteric glia, but not neurons (Gomes et al., 2009; Gulbransen et al., 2012). Collectively, these data are in support of an intermediate neuronal component between VPAC1 receptor activation and a subsequent P2Y1-mediated glial response, although other purinergic receptors may also be involved.

A number of neurons responded to the VPAC1 agonist after the glial activation. It is tempting to speculate that activated glia induce [Ca^2+^]_i_ transients in neurons to modulate activity within the enteric circuitry as shown in the CNS (Hansson and Rönnbäck, 2003). While there is no evidence for a physiological equivalent of the VPAC1 agonist, and VIP under control conditions does not stimulate glial [Ca^2+^]_i_ transients, it is conceivable that imbalances in the network may lead to the VPAC1 receptor-mediated pathway being preferentially activated. Alternatively, selective VPAC1-activation may occur in a physiological setting with localized VIP release, if VPAC1 and VPAC2 receptors are differentially expressed. Further, the observation of delayed glial responses to electrical stimulation revealed by inhibiting VPAC2 receptors suggests that these pathways may be activated by endogenous VIP to modulate signals from neurons to glia.

As VIP and VPAC1 receptors have an important role in neurogenic secretion (Banks et al., 2005; Xue et al., 2007; Fung et al., 2014), the neuron-to-glia signaling pathway we describe may contribute to secretomotor function. There are several reports suggesting that enteric glia are involved in the control of intestinal secretion. Mice administered a CNS gliotoxin, which also disrupted enteric glia, develop diarrhoea (Aikawa and Suzuki, 1985). On the other hand, treatment with the gliotoxin fluorocitrate did not result in changes in colonic ion transport (Nasser et al., 2006). Further, glial-specific connexin-43 knockout mice, in which the propagation of glial Ca^2+^ signals is disrupted, produce fecal pellets with higher water content (McClain et al., 2014), but this probably results from glial regulation of motility since chemogenetic activation of enteric glia *in vivo* did not affect fecal water content (McClain et al., 2015). MacEachern et al. (2015) have also reported a VIP-evoked neurogenic secretory response that was only observed when glial activity was perturbed in colitis. The mechanisms underlying these VIP-related (patho)physiological phenomena remain unknown and warrants more extensive investigation.

## Conclusion

**Figure 8:**
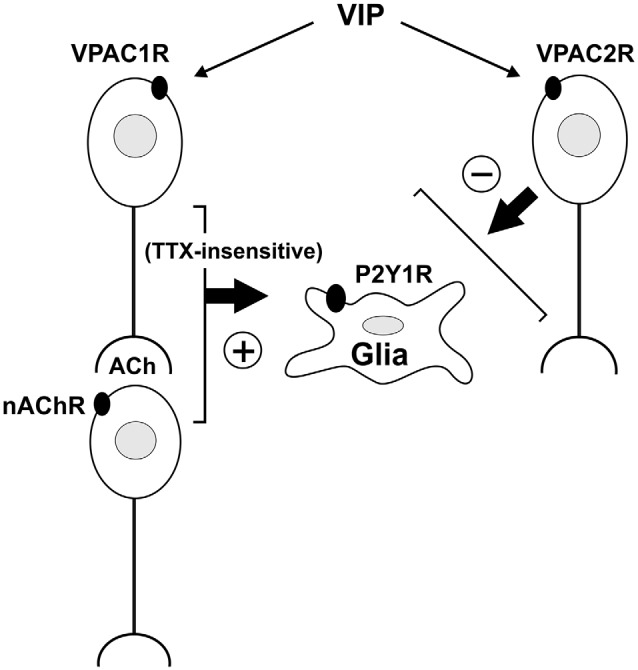
**A schematic of VIP-activated pathways in the mouse submucosal plexus determined using Ca^2+^ imaging**. This wiring diagram represents the most plausible neuro-glia circuit based on our experimental observations. VIP acting via VPAC1 and/or VPAC2 receptors elicits [Ca^2+^]_i_ transients in submucosal neurons. Neurons respond directly to VIP or secondarily via nicotinic transmission. Selective activation of VPAC1 receptors evokes glial [Ca^2+^]_i_ transients. This neuron to glia signaling pathway involves a TTX-insensitive mechanism. VPAC1 receptors expressed on cholinergic neurons and/or on CGRP^+^ nerve terminals may be involved. VIP also activates a VPAC2 receptor-mediated pathway that inhibits glial [Ca^2+^]_i_ responses. However, whether the VPAC2 receptor-expressing neuron inhibits glial responses directly or by inhibiting the VPAC1 receptor-mediated pathway, is yet to be determined. Note that the neurochemical identities of neurons involved are not illustrated for simplicity. Further, the possibility that VPAC1- and VPAC2-receptors are expressed on the same neuron, but activate different intracellular signaling pathways, cannot be excluded.

Our results show that activating VPAC1 receptors on cholinergic submucosal neurons stimulates purine release via a TTX-resistant mechanism to evoke [Ca^2+^]_i_ transients in glia. Inhibiting VPAC2 receptors also uncovered VIP-evoked glial responses. These experimental findings are summarized in a wiring diagram representing the most plausible neuro-glia circuit (Figure 8). Furthermore, we showed that electrically-evoked endogenous VIP release induced delayed glial responses when VPAC2 receptors were inhibited, thus confirming the presence of an intrinsic VIP/VPAC neuron-to-glia signaling pathway. Collectively, we have shown that stimulating neurally-released VIP can activate a secondary neuron, which may express VPAC1 and/or VPAC2 receptors, to modulate purine release onto glial cells. Thus, our data reveal a component of the enteric neuron-glia circuit that is regulated by VIP via a balanced activation of excitatory VPAC1- and inhibitory VPAC2-mediated pathways.

## Author Contributions

CF contributed to data collection and analyses and drafted the manuscript. WB contributed to data collection. CF, CC, WB, JPPF, JCB and PVB contributed to the experimental design and interpretation of the data. JCB, JPPF, WB, CC and PVB contributed to the conception of the project. All authors contributed to editing and revising the manuscript. All authors read and approved the final manuscript.

## Funding

This work was funded by grants from (Fonds voor Wetenschappelijk Onderzoek, FWO) grants to WB and PVB (G.0501.10 and G.0921.15) and NHMRC Australia (1006453) and the Australian Research Council (DP130101596) to JCB. CC and WB are postdoctoral fellows of the FWO.

## Conflict of Interest Statement

The authors declare that the research was conducted in the absence of any commercial or financial relationships that could be construed as a potential conflict of interest.
